# A Hypomorphic Mutant of PHD Domain Protein Male Meiocytes Death 1

**DOI:** 10.3390/genes12040516

**Published:** 2021-04-01

**Authors:** Bing Liu, Chunlian Jin, Nico De Storme, Sébastien Schotte, Cédric Schindfessel, Tim De Meyer, Danny Geelen

**Affiliations:** 1 College of Life Sciences, South-Central University for Nationalities, Wuhan 430074, China; 2 Unit HortiCell, Department of Plants and Crops, Faculty of Bioscience Engineering, Ghent University, 9000 Ghent, Belgium; chunlian.jin@ugent.be (C.J.); nico.destorme@kuleuven.be (N.D.S.); Sebastien.Schotte@UGent.be (S.S.); Cédric.Schindfessel@UGent.be (C.S.); 3 Division of Crop Biotechnics, Department of Biosystems, KU Leuven, 3001 Leuven, Belgium; 4 Department of Data Analysis and Mathematical Modelling, Ghent University, Coupure Links, 9000 Ghent, Belgium; Tim.DeMeyer@UGent.be

**Keywords:** meiosis, meiotic restitution, meiotic cytokinesis, unreduced gametes, MMD1

## Abstract

Meiosis drives reciprocal genetic exchanges and produces gametes with halved chromosome number, which is important for the genetic diversity, plant viability, and ploidy consistency of flowering plants. Alterations in chromosome dynamics and/or cytokinesis during meiosis may lead to meiotic restitution and the formation of unreduced microspores. In this study, we isolated an *Arabidopsis* mutant *male meiotic restitution 1* (*mmr1*), which produces a small subpopulation of diploid or polyploid pollen grains. Cytological analysis revealed that *mmr1* produces dyads, triads, and monads indicative of male meiotic restitution. Both homologous chromosomes and sister chromatids in *mmr1* are separated normally, but chromosome condensation at metaphase I is slightly affected. The *mmr1* mutant displayed incomplete meiotic cytokinesis. Supportively, immunostaining of the microtubular cytoskeleton showed that the spindle organization at anaphase II and mini-phragmoplast formation at telophase II are aberrant. The causative mutation in *mmr1* was mapped to chromosome 1 at the chromatin regulator *Male Meiocyte Death 1* (*MMD1*/*DUET*) locus. *mmr1* contains a C-to-T transition at the third exon of *MMD1*/*DUET* at the genomic position 2168 bp from the start codon, which causes an amino acid change G618D that locates in the conserved PHD-finger domain of histone binding proteins. The F1 progenies of *mmr1* crossing with knockout *mmd1*/*duet* mutant exhibited same meiotic defects and similar meiotic restitution rate as *mmr1*. Taken together, we here report a hypomorphic *mmd1*/*duet* allele that typically shows defects in microtubule organization and cytokinesis.

## 1. Introduction

Most higher plants, especially for angiosperms, have undergone at least one round of whole genome duplication (WGD) in evolution [1,2]. Formation and fusion of diploid or polyploid gametes are considered the primary route to plant polyploidization [3,4]. Alterations in one or more meiosis processes, including omission of meiotic cell cycle, defective chromosome segregation, spindle misorientation, and/or incomplete meiotic cytokinesis, are the common mechanisms leading to the generation of unreduced gametes through restitution of meiotic cell division [5,6,7,8,9,10]. In concern of the fundamental significance and practical utilization for polyploid crop breeding, it is of particular importance to uncover the genetic factors involved in unreduced gamete formation.

In *Arabidopsis*, at least 23 genes have been identified, which, in case of dysfunction, cause meiotic restitution (Table 1). Typically, functional mutations of the genes that control meiotic cell cycle transition, such as *OSD1*/*GIG1* and *TAM*/*CYCA1;2*, terminate the meiosis program prematurely, thereby inducing the production of 2n eggs and 2n spores [11]. Moreover, alterations of the three-dimensional positioning of spindles may result in unreduced gamete formation. The mechanism asserting the perpendicular position of the spindles is lost in *parallel spindle1 (ps1)* and *jason* mutants, which results in partial or complete fusion of the spindles [12,13]. Male meiotic cytokinesis in *Arabidopsis* is regulated by a mitogen-activated protein kinase (MAPK) signaling cascade composed of TES/STUD/AtNACK2-ANPs-MKK6/ANQ-MPK4. Loss of function of any member in this module leads to defects in male meiotic cytokinesis and the formation of pollen grains with an increased ploidy level [14,15,16,17,18]. Remarkably, the chromatin regulator Male Meiocyte Death 1 (MMD1/DUET), a PHD-finger protein that reads and binds with H3K4methylation sites, plays an important role in regulating multiple processes in *Arabidopsis* male meiosis [1,19,20,21]. MMD1/DUET regulates the expression of meiotic genes, including *TDM1*, *JASON*, and *CAP-D,3,* through binding to the H3K4me2/3 sites in the promoter regions [22,23]. The null *mmd1*/*duet* mutant displays chromosome de-condensation at metaphase I, altered meiosis progression, irregular spindle organization at anaphase II, and aberrant phragmoplast formation at telophase II, which lead to meiocyte cell death, meiotic restitution, and impaired plant fertility [22,23,24,25].

In search of genetic factors that contribute to ploidy consistency in meiosis, we previously performed a forward genetic screen of ethyl methanesulfonate (EMS)-mutagenized *Arabidopsis thaliana* Col-0 plants for mutants that produce over-sized pollen grains [13]. Here, the mutant *male meiotic restitution 1* (*mmr1*) is described to consistently produce a relatively small fraction of unreduced pollen grains. The *mmr1* mutant undergoes meiotic restitution and produces diploid and/or polyploid microspores. Meanwhile, *mmr1* displays a mildly impacted chromosome condensation at metaphase I. Furthermore, the organization of spindles and phragmoplast in late meiosis II meiocytes of *mmr1* are interfered, which results in defective cytokinesis. The causative point mutation in *mmr1* was mapped to the third exon of *MMD1*/*DUET* within the conserved PHD domain. Overall, we here describe an allelic and hypomorphic mutant of *Arabidopsis* chromatin regulator *MMD1*/*DUET*.

## 2. Materials and Methods

### 2.1. Plant Materials and Growth Conditions

*Arabidopsis thaliana* wild-type accession Col-0 was used in this study. EMS-mutagenized Col-0 seeds were obtained from the Nottingham Arabidopsis Stock Centre. *Arabidopsis pWOX2::CENH3-GFP* [43] and *mmd1*/*duet* [22] were previously described. Seeds were germinated on K1 medium for 6 to 8 days, and seedlings were transferred to soil and cultivated in growth chambers at 12 h light/12 h night, 20 °C, and 70% humidity. Upon bolting, the photoperiod was changed to a 16 h day/8 h night regime.

### 2.2. Cytology and Microscopy

Pollen 4’,6-diamidino-2-phenylindole (DAPI) staining, callosic cell wall staining, and analysis of the male meiotic products (tetrad-stage analysis by orcein and DAPI staining) were performed as described [21]. Sperm formation and chromosome counting was performed using the fluorescent marker *pWOX2::CENH3-GFP* and *pMGH3::H2B-GFP* lines. Spores were released in a 0.05 M phosphate buffer (pH 7.0) containing 0.5% Triton X-100 (*v*/*v*). Meiotic chromosomes were visualized following meiotic chromosome spreading and microtubules using immunostaining as described [21,44]. The assessment of unreduced pollen and microspores was performed by comparing the size to the haploid pollen (diameter >28 µm) or by counting the number of nuclei [45]. Bright-field and fluorescence microscopy were performed using an Olympus IX81 inverted fluorescence microscope equipped with an X-Cite Series 120Q UV lamp and an Olympus XM10 camera. Bifluorescent images and Z-stacks were processed using ImageJ. Brightness and contrast settings were adjusted using Photoshop CS6.

### 2.3. Identification of the Causative Mutation

The causative mutation in *mmr1* was identified through bulk segregate analysis of progeny from a Col-0/L*er* hybrid. The microsporogenesis of the F1 progenies obtained by homozygous *mmr1* plants (female) crossing with L*er* plants (male) were checked under microscope, and the genotype of the F1 progenies was examined by PCR using the primers listed in Supplementary Table S1. F2 population was collected by F1 selfing. Then, 1100 F2 individuals were checked and the samples showing meiotic restitution/samples phenocopied wild-type plants = 1:3, confirming that the meiotic restitution in *mmr1* is caused by a single recessive mutation. F2 plants showing more than 5% oversized pollen grains were selected and genomic DNA was isolated and pooled for sequencing.

## 3. Results

### 3.1. Isolation of mmr1 That Produces Diploid and/or Polyploid Pollen Grains

**Figure 1:**
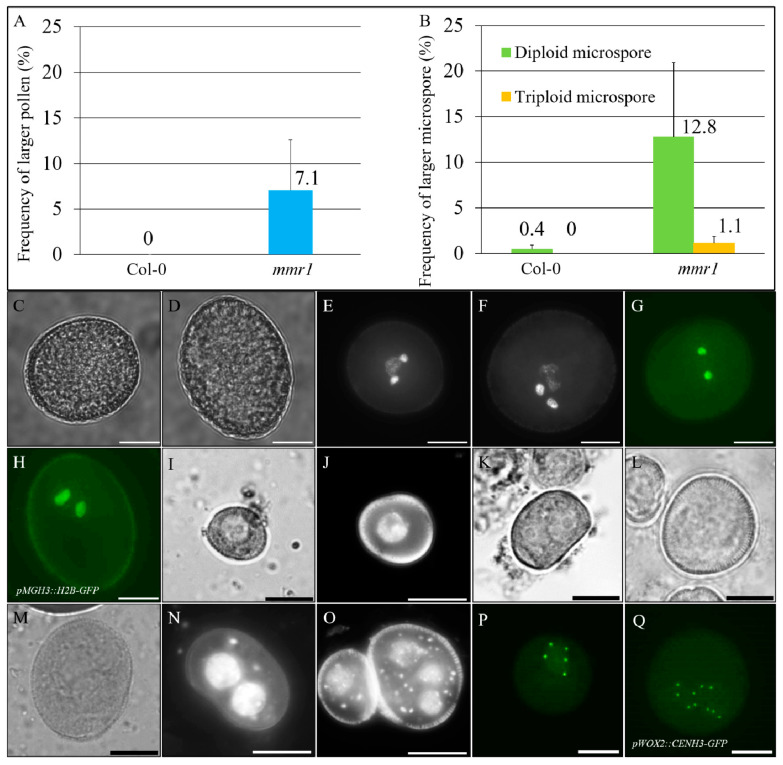
*Male meiotic restitution 1* (*mmr1*) generates unreduced gametes. (**A**,**B**) Histograms showing the frequency of enlarged pollen grains (**A**) and diploid and/or triploid unicellular stage microspores (**B**) in *mmr1*. Error bars represent standard deviation of the mean values. Numbers indicate the average rate of the corresponding phenotypes. Three bio-replicates were analyzed. (**C**,**D**) Bright field images of mature pollen grains in Col-0 (**C**) and *mmr1* (**D**). (**E**,**F**) DAPI-stained mature pollen grains in Col-0 (**E**) and *mmr1* (**F**). (**G**,**H**) Mature pollen grains expressing *pMGH3::H2B-GFP* in Col-0 (**G**) and *mmr1* (**H**). (**I**,**J**) Unicellular stage microspores by bright field imaging (**I**) and DAPI-staining (**J**) in Col-0. (**K**–**O**) Unicellular stage microspores by bright field imaging (**K,** diploid; **L**, triploid; **M**, tetraploid) and DAPI staining (**N**, diploid; **O**, triploid) in *mmr1*. (**P**,**Q**) Unicellular stage microspores expressing *pWOX2::CENH3-GFP* in Col-0 (**P**) and *mmr1* (**Q**). Scale bars = 10 µm.

Previously, we screened an M2 Col-0 *Arabidopsis* EMS-mutagenized population for mutants that produce pollen grains with enlarged sizes. In this screen, we identified the *male meiotic restitution 1* (*mmr1*) that produces approximately 7.1% over-sized pollen grains (Figure 1A,C, Col-0 and D, *mmr1*). 4′,6-diamidino-2-phenylindole (DAPI) staining revealed that the enlarged pollen contained larger sperms indicative of a higher DNA content (Figure 1E, Col-0; F, *mmr1*; Supplementary Figure S1A–C). In support, *mmr1* plants expressing *pMGH3::H2B-GFP* that labels sperm nuclei [46] exhibited larger H2B fluorescent signals (Figure 1G, Col-0; H, *mmr1*; Supplementary Figure S1D–F). Occasionally, the enlarged *mmr1* pollen grains were found to contain more than two sperm (Supplementary Figure S1C,F), indicating a potential defect in cell division or chromosome dynamics. Unicellular stage microspores were next examined, which showed that *mmr1* generated larger microspores with multiple nuclei (Figure 1I,J, Col-0; K–O, *mmr1*), suggesting that the enlarged pollen grains originated from the over-sized microspores. We then introduced *pWOX2::CENH3-GFP* into the *mmr1* mutant to determine the exact chromosome number of the enlarged gametes [43]. Most unicellular stage microspores from wild-type showed five CENH3 foci, which represent halved chromosome counts (Figure 1B,P). In contrast, the enlarged *mmr1* microspores contained either 10 (12.8%) or 15 (1.1%) CENH3 foci (Figure 1B,Q; Supplementary Figure S2A,B), indicating these over-sized microspores were diploid and/or triploid, respectively. Meanwhile, the *mmr1* mutant showed normal silique development (Supplementary Figure S3A,B) but yielded less seeds than wild-type plants (Supplementary Figure S3C). These findings suggest that *mmr1* produces unreduced gametes and has an interfered fertility.

### 3.2. mmr1 Undergoes Meiotic Restitution

**Figure 2:**
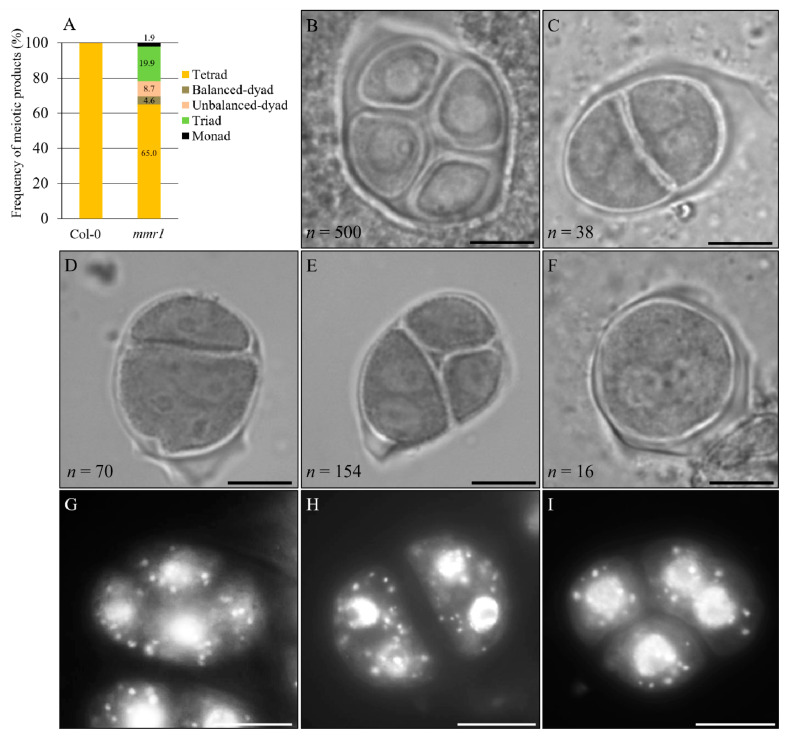
*mmr1* undergoes meiotic restitution. (**A**). Histogram showing the frequency of tetrad-stage meiotic products generated by *mmr1* plants. Numbers indicate the frequency of the meiotic products. (**B**) Normal tetrad in Col-0. (**C**–**F**) Balanced dyad (**C**), unbalanced dyad (**D**), triad (**E**), and monad (**F**) in *mmr1*. *n* represents the number of cells used for quantification, and five biological individuals were analyzed. (**G**–**I**) DAPI-stained tetrad stage meiocytes in Col-0 (**G**) and *mmr1* (**H**,**I**). Scale bars = 10 µm.

The formation of unreduced microspores suggests that meiotic restitution occurred in the microsporogenesis of *mmr1*. We hence analyzed and quantified tetrad-stage meiocytes in *mmr1* using orcein staining. Wild-type plants consistently generated balanced tetrads that contained four haploid spores with each harboring one nucleus (Figure 2A,B). However, *mmr1* plants were found to produce approximately 35.0% meiotic restituted products (Figure 2A) with 19.9% triads (Figure 2A,E), 8.7% unbalanced dyads (Figure 2A,D), 4.6% balanced dyads (Figure 2A,C) and 1.9% monads (Figure 2A,F), respectively. DAPI staining of tetrad-stage meiocytes confirmed the formation of dyad and triad with more than one nucleus per spore, which represented occurrence of meiotic restitution in the *mmr1* mutant (Figure 2G, Col-0; H,I, *mmr1*).

**Figure 3:**
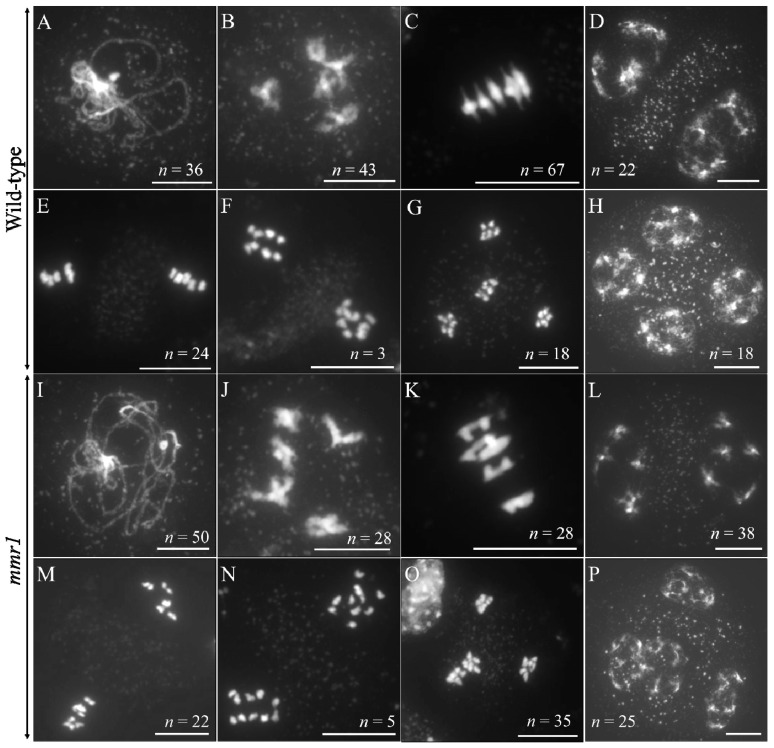
Meiotic spreading of wild-type Col-0 and *mmr1* plants. (**A**–**H**) DAPI-stained chromosomes at pachytene (**A**), diakinesis (**B**), metaphase I (**C**), interkinesis (**D**), metaphase II (**E**), anaphase II (**F**), telophase II (**G**), and tetrad stage (**H**) in Col-0. (**I**–**P**) DAPI-stained chromosomes at pachytene (**I**), diakinesis (**J**), metaphase I (**K**), interkinesis (**L**), metaphase II (**M**), anaphase II (**N**), telophase II (**O**), and tetrad stage (**P**) in *mmr1*. *n* indicates the number of observed cells at corresponding stages. Scale bars = 10 µm.

Meiotic spreading was performed to monitor the chromosome behaviors in *mmr1* meiosis (Figure 3). In wild-type plants, homologous chromosomes were fully paired at pachytene (Figure 3A), and five pairs of bivalents occurred at diakinesis (Figure 3B). No obvious defect was observed in *mmr1* meiocytes at these stages, indicating normal homolog synapsis and crossover formation (Figure 3I,J). At metaphase I, most bivalents in the wild-type showed chromosomes under tension (ratio of bivalents with tension/bivalents without tension = 2.5), aligned at the equatorial plate (Figure 3C). In *mmr1*, however, more bivalents displayed an aberrant shape at metaphase I and lacked the typical thin threads at either side that result from the pulling force at the kinetochore (bivalents with tension/bivalents without tension = 1.5) (Figure 3K). The minor difference of metaphase I chromosomes suggested that the *mmr1* mutation has a mild impact on chromosome condensation. *mmr1* chromosomes behaved normally from the interkinesis to metaphase II stages as control (Figure 3D,E, Col-0; L,M, *mmr1*). At anaphase II, the sister chromatids in *mmr1* separated, indicating regular sister chromatid cohesion dynamics (Figure 3F, Col-0; N, *mmr1*). At telophase II, however, unlike Col-0 meiocytes that displayed four balanced isolation of chromosome sets, *mmr1* occasionally showed clustered chromosomes representing restitution configurations (Figure 3G, Col-0; O, *mmr1*), which consequently led to adjacent or fused nuclei at tetrad stage (Figure 3H, Col-0; P, *mmr1*; Supplementary Figure S4A–C). These data indicate that although both homologous chromosomes and sister chromatids in the *mmr1* mutant were able to segregate, positioning of haploid chromosome sets at telophase II was somehow interfered, which implied an alteration in proper spindle orientation and/or cytokinesis.

### 3.3. Meiotic Cytokinesis Is Defective in mmr1

**Figure 4:**
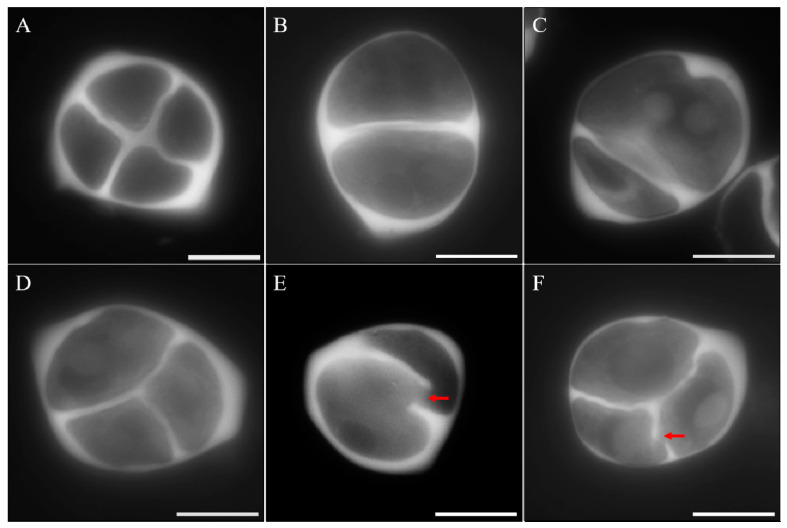
Meiotic cell formation is defective in *mmr1*. (**A**) Tetrad with a complete meiotic cell wall in the wild-type. (**B**–**F**) Balanced dyad (**B**), unbalanced dyads (**C**,**E**), and triads (**D** and **F**) in *mmr1*. Red arrows indicate the interruptions in meiotic cell walls of *mmr1*. Scale bars = 10 µm.

To determine whether meiotic restitution in *mmr1* was caused by a defect in cytokinesis, we analyzed meiotic cell walls using aniline blue staining that specifically marks callose. Tetrads in the wild-type plants generated a cross-shaped cell wall configuration, indicating four haploid spores (Figure 4A). In contrast, *mmr1* meiocytes were observed to have one or more cell walls omitted, displaying balanced dyads (Figure 4B), unbalanced dyads (Figure 4C,E) or triads (Figure 4D,F). Moreover, interrupted cell walls were occasionally observed (Figure 4E,F, see red arrows). These figures indicate that *mmr1* is defective for generating complete meiotic cell walls during cytokinesis.

### 3.4. Altered Spindle Orientation and Phragmoplast Formation in mmr1

**Figure 5:**
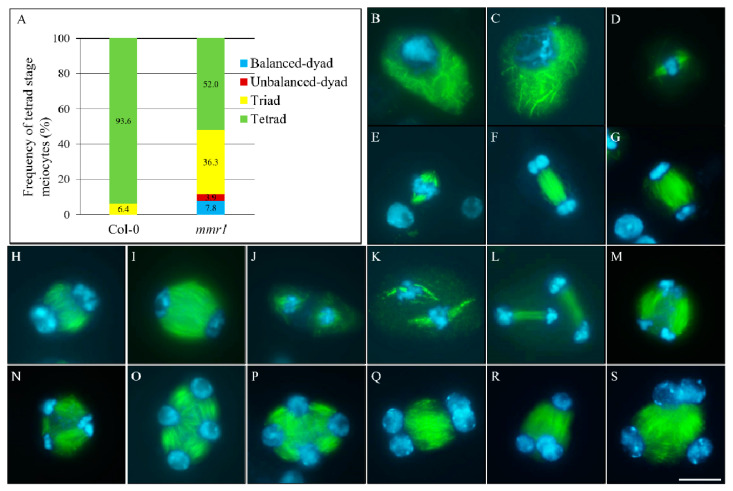
Microtubular cytoskeleton structures in *mmr1* meiocytes. (**A**) Histogram showing the frequency of tetrad stage meiocytes in *mmr1*. Numbers indicate the frequency of the corresponding phenotypes. (**B**–**S**) Prophase I- (**B**,**C**), metaphase I- (**D**,**E**), anaphase I- (**F**,**G**), interkinesis- (**H**,**I**), metaphase II- (**J**,**K**), telophase II- (**L**–**N**), and tetrad-stage (**O**–**S**) meiocytes in Col-0 (**B**,**D**,**F**,**H**,**J**,**L**,**O**) and the *mmr1* mutant (**C**,**E**,**G**,**I**,**K**,**M**,**N**,**P**–**S**). Green, α-tubulin; cyan, DAPI. Scale bar = 10 µm.

Construction of meiotic cell walls relies on the organization of the microtubular cytoskeleton surrounding chromosomes or nuclei [21]. Immunostaining of α-tubulin was therefore applied to elucidate whether the meiotic cytokinesis defects in *mmr1* was caused by any alteration in microtubules. From prophase I to metaphase II, *mmr1* did not show any obvious difference in microtubular network formation, as in wild-type plants (Figure 5B,D,F,H,J, Col-0; C,E,G,I,K, *mmr1*). Successfully generated spindles at metaphase I and II supported the observation that both chromosome segregation cycles were not influenced in *mmr1* (Figure 5E,K). However, at telophase II, the orientation of phragmoplast in *mmr1* displayed either parallel or tripolar configuration (Figure 5L, Col-0; M,N, *mmr1*; Supplementary Figure S5A,B). In wild-type and most *mmr1* tetrad stage meiocytes, radial microtubule arrays (RMAs) were organized into mini-phragmoplasts between the separated nuclei, contributing to tetrad formation (Figure 5O, Col-0; P, *mmr1*). Approximately 48.0% tetrad stage meiocytes in *mmr1*, however, showed omission of one or more RMAs between the separated nuclei (Figure 5A; Supplementary Table S2), which generated triads (36.3%) (Figure 5S; Supplementary Figure S5G,H), balanced-dyads (7.8%) (Figure 5Q; Supplementary Figure S5C,D) and unbalanced dyads (3.9%) (Figure 5R; Supplementary Figure S5E,F). In addition, the telophase II phragmoplasts in *mmr1* were broader than in Col-0 and contained microtubules that were not in line with the axes across the nuclei (Figure 5L, Col-0; M,N, and Supplementary Figure S5G, *mmr1*). Meanwhile, the microtubules of RMAs were also less compacted than in Col-0 (Figure 5O, Col-0; P–S and Supplementary Figure S5C–H, *mmr1*). Taken together, these findings revealed defective spindle orientation, and formation and/or organization of phragmoplasts at meiosis II in the *mmr1* mutant.

### 3.5. mmr1 Carries a Point Mutation in the PHD-Finger Domain of MMD1/DUET

**Figure 6:**
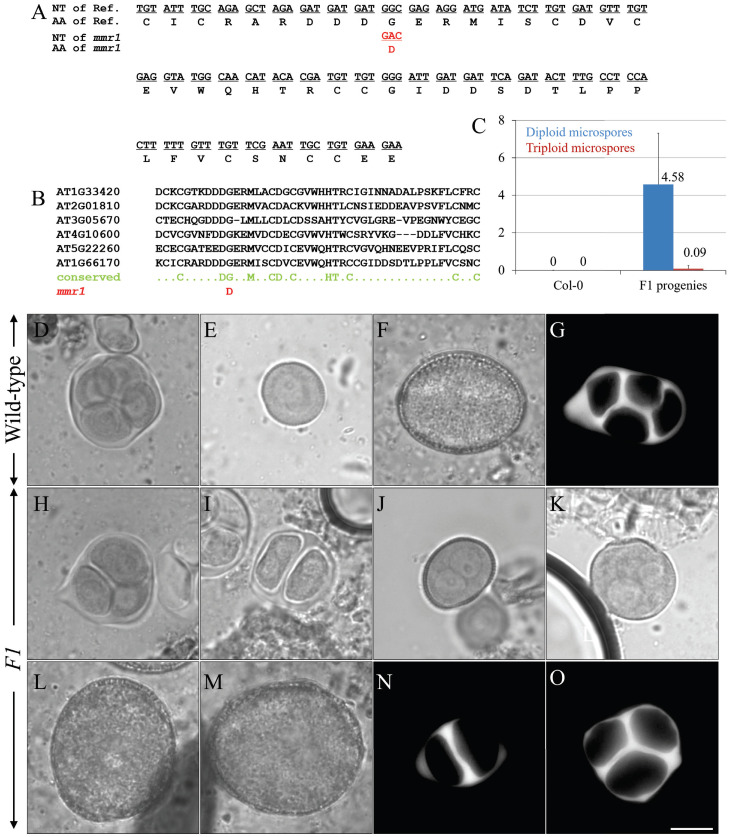
*mmr1* is an allelic mutant of *MMD1/DUET*. (**A**) Figure showing the nucleotide (NT) sequence and amino acid sequence of the PHD domain in the *MMD1/DUET* gene and protein, and the changes of that in *mmr1*. (**B**) Figure showing the conservation of the amino acid in the PHD domain in multiple histone binding proteins, and the point mutation-caused amino acid replacement in that of *mmr1*. (**C**) Histogram showing the frequency of diploid and triploid microspores produced by *mmr1*/*mmd1* F1 progenies. Nine individuals were quantified. Numbers indicate the frequency of diploid and/or triploid microspores. (**D**,**H**,**I**) Orcein-stained tetrads in the Col-0 plants (**D**), and triad (**H**) and dyad (**I**) in the *mmr1*/*mmd1* F1 progenies. (**E**,**J**,**K**) Haploid unicellular stage microspore in the Col-0 plants (**E**), and diploid (**J**) and polyploid (**K**) unicellular-stage microspores in the *mmr1*/*mmd1* F1 progenies. (**F**,**L**,**M**) Haploid pollen grain in the Col-0 plants (**F**), and enlarged pollen grains in the *mmr1*/*mmd1* F1 progenies (**L**,**M**). (**G**,**N**,**O**) Aniline blue-stained meiotic cell walls in the Col-0 plants (**G**) and in the *mmr1*/*mmd1* F1 progenies (**N**,**O**). Scale bar = 10 µm.

To identify the causative mutation in the *mmr1* mutant, we performed bulk segregant analysis combined with whole genome sequencing. F1 progenies from the intercrossing between *mmr1* mutant and wild-type Landsberg *erecta* (L*er*) was genotyped using simple sequence length polymorphism (SSLP) primers to check Col0/Ler heterozygosity (Supplementary Figure S6). All of the F1 individuals displayed normal male meiotic cytokinesis and produced normal-sized male gametes (Supplementary Figure S7A–C), suggesting a recessive mutation in *mmr1*. The phenotypic screening of 1100 F2 Col/L*er* descendants resulted in 250 individuals producing unreduced microspores, and DNA from individuals were pooled and sequenced. A 26 Mb region on chromosome 1 was enriched for Col-0 SNPs (Supplementary Figure S8). Several genes within this region carried mutations, but the candidate gene *MMD1*/*DUET* (AT1G66170) was the only meiosis-specific one. *mmr1* was found to carry a single C-to-T transition at the genomic position 2168 bp from the start codon of *MMD1*/*DUET*, leading to an amino acid change G618D (Figure 6A; Supplementary Table S3). The G618D amino acid change was located within the plant homeodomain (PHD) of MMD1/DUET, which has been found in many chromatin regulatory factors and conserved in histone binding proteins (Figure 6B) [24]. A complementation test was performed by crossing homozygous *mmr1* mutant with homozygous *mmd1* null mutant using *mmr1* as the pollen donor. All checked F1 progenies produced unreduced microspores and pollen, and showed defects in meiotic cytokinesis (Figure 6C–G, Col-0; H–O, *mmr1*/*mmd1* F1 progenies), which indicates that *mmr1* is allelic to *MMD1/DUET*. Since *mmr1* produced a small population of unreduced microspores, it is thus a hypomorphic *mmd1*/*duet* allele.

## 4. Discussion

In this study, we reported the identification of a recessive mutant *mmr1* that consistently produces a small fraction (around 5%–10%) of unreduced pollen grains. The phenotype is caused by a single amino acid change in the PHD domain of MMD1/DUET, a chromatin regulator that is specifically expressed in male meiocytes and functions in male meiosis [24,25]. Ds transposon insertions in the exon of *MMD1/DUET* lead to *MMD1/DUET* null mutants, which display strong male sterility due to various defects in meiosis that culminate into meiocyte cell death [24,25]. Male meiosis in *MMD1/DUET* is initiated normally, forming meiocytes that progress up to pachytene as in the wild-type. During the subsequent steps in the meiosis program, two cycles of chromosome condensation take place. In *mmd1/duet*, diakinesis chromosomes are paired into bivalents and appear less compact and show inter-bivalent interactions [24,25,47], which were, however, not observed in *mmr1*. In *mmr1*, an alteration in chromosome structure was observed at metaphase I, when bivalents are under tension from kinetochore microtubule pulling forces. In the wild-type, the kinetochores are slightly pulled apart, which is evident from the thin DAPI-stained threads perpendicular to the equatorial plane. These extensions are also absent, or at least far less pronounced, in null *mmd1/duet* [23]. In *mmr1*, the altered chromosome condensation in metaphase I is not as severe as in the null *mmd1*/*duet* mutant, suggesting that *mmr1* is hypomorphic. The reduction in fertility of *mmr1* is very mild compared to *mmd1/duet*, in line with the occurrence of cytological defects in only a subpopulation of the meiocytes and microspores. The *mmr1* mutation therefore has a minor impact on the functioning of *MMD1/DUET*.

The G618D amino acid change in *mmr1* is located within the PHD domain of MMD1/DUET, which forms an aromatic cage, required for the specific binding to H3K4me2 [22,23,47]. MMD1/DUET may bind a wider range of histone peptides, as a recombinant peptide encompassing the MMD1/DUET PHD finger (601 to 659) was shown to bind the histone peptides H3K4me2/me3, H3K9me2, and H3S10 [23]. The G618D amino acid change in the *mmr1* allele introduces a negative charge in the PHD finger, modifying its electrostatic balance and likely changing the strength and/or specificity of histone binding. As *mmr1* displays a hypomorphic phenotype, we speculate that the *mmr1* allele has the same histone specificity but with slightly weaker binding affinity. The PHD domain is of critical importance to MMD1/DUET function, as PHD deletion mutants and selected PHD amino acid changes show the same sterility phenotype as a knockout line [23,47]. Andreuzzi et al. (2015) [22] reported on a hypomorphic MMD1/DUET PHD finger mutation that causes the formation of a low frequency of enlarged pollen grains (approximately 5%) reminiscent to *mmr1* [22]. The reported hypomorphic mutant appeared to be defective specifically in meiosis II spindle and RMA organization [22]. Chromosome condensation, M II phragmoplast, and RMA organization are affected in *mmr1*, suggesting that the G618D amino acid change interferes with MMD1/DUET functioning during both meiosis I and II. The mild alteration in chromosome condensation at metaphase I in *mmr1* suggests that the point mutation G618D is more related to MMD1/DUET function in phragmoplast organization.

## 5. Conclusions

MMD1/DUET has been shown to profoundly modulate gene expression in anthers and seems to do this differentially during early stage and late stage of meiosis [23]. Expression of the meiotic cell cycle regulator *TDM* and the meiosis spindle II organizer *JASON* is severely reduced in *duet*, in line with the defects in spindle position and cytokinesis [22]. MMD1/DUET also directly regulates the expression of the condensin genes *CAP-D2, CAP-D3, CAP-H,* and *CAP-H2*. The *cap-d3* mutants show a male meiotic chromosome condensation phenotype such as *mmd1*/*duet*, indicating that the prophase I chromosome condensation phenotype is due the misexpression of *CAP-D3* in *MMD1/DUET* [23]. Since *mmr1* shows a very mild phenotype in metaphase I chromosome condensation and a low frequency of meiotic restitution, we speculate that a reduction in the expression of *CAP-D3* and *Jason* is minor and only occasionally drops below a threshold to generate the observed defects in chromosome condensation and cytokinesis. The isolated *mmr1* mutant in this report thus provides a tool to analyze the function of regions within the PHD domain in MMD1/DUET with a specific correlation to telophase II and cytokinesis.

## Figures and Tables

**Table 1:** Genes, dysfunction of which may cause male meiotic restitution in *Arabidopsis thaliana*.

Genes	Gene Function	Reference
*AESP*	Proper disjunction of chromosomes	(Liu and Makaroff, 2006) [26]
*AFH14*	Organization of microtubule and microfilament arrays	(Li et al., 2010) [27]
*ANQ1/MKK6*	Male meiotic cytokinesis	(Takahashi et al., 2010) [16]
*CALS5*	Callose synthase	(Dong et al., 2005) [28]
*CDM1*	Callose metabolism	(Lu et al., 2014) [29]
*CYCA1;2/TAM*	Meiosis II cell cycle transition	(d’Erfurth et al., 2010) [11]
*MMD1/DUET*	Chromatin condensation, spindle and phragmoplast formation	(Andreuzza et al., 2015; Wang et al., 2016) [22,23]
*DYAD/SWITCH1*	Meiotic chromosome organization	(Ravi et al., 2008) [30]
*GSL1* and *5*	Callose wall formation	(Enns et al., 2005) [31]
*JASON*	Meiosis II spindle orientation	(Brownfield et al., 2015; De Storme and Geelen, 2011) [13,32]
*KINESIN-12*	Polymerization of phragmoplast microtubules	(Liu et al., 2010; Oh et al., 2014) [33,34]
*MPK4*	Male meiotic cytokinesis	(Kosetsu et al., 2010; Zeng et al., 2011) [18,35]
*MS4*	Meiosis progression	(Chaudhury et al., 1994) [36]
*MYB33/65*	GA signaling downstream transcription regulators	(Liu et al., 2017; Millar and Gubler, 2005) [37,38]
*OSD1*	Meiosis cycle transition	(Cromer et al., 2012; d’Erfurth et al., 2009) [19,39]
*PANS1*	Centromeric cohesion maintenance of sister chromatid in meiosis II	(Zamariola et al., 2014) [40]
*AtPS1*	Meiosis II spindle orientation	(Brownfield et al., 2015; d’Erfurth et al., 2008) [12,32]
*RGA* and *GAI*	GA signaling repressors	(Liu et al., 2017) [37]
*RSW4*	RMA organization	(Yang et al., 2011) [41]
*SDS*	Homolog synapsis and recombination	(Azumi et al., 2002) [20]
*SHUGOSHIN 1/2*	Centromeric cohesion maintenance at anaphase I	(Zamariola et al., 2013; Zamariola et al., 2014) [40,42]
*TES2*	Male meiotic cytokinesis	(Spielman et al., 1997; Yang et al., 2003a) [15,17]
*TIO*	Male meiotic cytokinesis	(Oh et al., 2014) [34]

## Data Availability

No new data were created or analyzed in this study. Data sharing is not applicable to this article.

## Supplementary Materials

The following are available online at https://www.mdpi.com/article/10.3390/genes12040516/s1, Supplementary Figure S1. *mmr1* produces enlarged pollen grains with increased DNA content**;** Supplementary Figure S2. *mmr1* produces diploid or polyploid microspores; Supplementary Figure S3. *mmr1* displays a mildly reduced fertility; Supplementary Figure S4. Tetrad-stage meiocytes in the *mmr1* mutant stained by DAPI; Supplementary Figure S5. *mmr1* exhibits defective phragmoplasts and radial microtubule array (RMA); Supplementary Figure S6. Genotyping of the F1 individual obtained by crossing between homozygous *mmr1* mutant and wild-type L*er* plants using simple sequence length polymorphism (SSLP) marker primer F3L12; Supplementary Figure S7. Phenotyping of F1 plants obtained by crossing homozygous *mmr1* mutant with wild-type L*er* plants; Supplementary Figure S8. Whole genome sequencing analysis of pooled *mmr1*/L*er* F2 plants showing the large pollen phenotype; Supplementary Table S1. Primer for genotyping of F1 progenies (*mmr1* crossing with wild-type L*er*); Supplementary Table S2. Quantification of *mmr1* meiotic restitution by monitoring microtubules; Supplementary Table S3. Mutation of *mmr1* at the *MMD1*/*DUET* locus.

## References

[1] Del Pozo J.C., Ramirez-Parra E. (2015). Whole genome duplications in plants: An overview from Arabidopsis. J. Exp. Bot..

[2] Ren R., Wang H.F., Guo C.C., Zhang N., Zeng L.P., Chen Y.M., Ma H., Qi J. (2018). Widespread whole genome duplications contribute to genome complexity and species diversity in angiosperms. Mol. Plant.

[3] Bretagnolle F., Thompson J.D. (1995). Gametes with the somatic chromosome number: Mechanisms of their formation and role in the evolution of autopolyploid plants. New Phytol..

[4] Ramsey J., Schemske D.W. (1998). Pathways, mechanisms, and rates of polyploid formation in flowering plants. Annu. Rev. Ecol. Syst..

[5] Brownfield L., Kohler C. (2011). Unreduced gamete formation in plants: Mechanisms and prospects. J. Exp. Bot..

[6] De Storme N., Geelen D. (2013). Sexual polyploidization in plants--cytological mechanisms and molecular regulation. New Phytol..

[7] De Storme N., Geelen D., Pradillo M., Heckmann S. (2020). Induction and characterization of diploid pollen grains in Arabidopsis thaliana. Plant Meiosis: Methods and Protocols.

[8] Kreiner J.M., Kron P., Husband B.C. (2017). Evolutionary dynamics of unreduced gametes. Trends Genet..

[9] Loginova D.B., Silkova O.G. (2017). Mechanisms of Unreduced Gamete Formation in Flowering Plants. Russ. J. Genet..

[10] Mason A.S., Pires J.C. (2015). Unreduced gametes: Meiotic mishap or evolutionary mechanism?. Trends Genet..

[11] d’Erfurth I., Cromer L., Jolivet S., Girard C., Horlow C., Sun Y., To J.P.C., Berchowitz L.E., Copenhaver G.P., Mercier R. (2010). The cyclin-A CYCA1;2/TAM is required for the meiosis I to meiosis II transition and cooperates with OSD1 for the prophase to first meiotic division transition. PLoS Genet..

[12] d’Erfurth I., Jolivet S., Froger N., Catrice O., Novatchkova M., Simon M., Jenczewski E., Mercier R. (2008). Mutations in AtPS1 (Arabidopsis thaliana Parallel Spindle 1) Lead to the Production of Diploid Pollen Grains. PLoS Genet..

[13] De Storme N., Geelen D. (2011). The Arabidopsis mutant jason produces unreduced first division restitution male gametes through a parallel/fused spindle mechanism in meiosis II. Plant Physiol..

[14] Hülskamp M., Parekh N.S., Grini P., Schneitz K., Zimmermann I., Lolle S.J., Pruitt R.E. (1997). The STUD gene is required for male-specific cytokinesis after telophase II of meiosis in Arabidopsis thaliana. Dev. Biol..

[15] Spielman M., Preuss D., Li F.L., Browne W.E., Scott R.J., Dickinson H.G. (1997). TETRASPORE is required for male meiotic cytokinesis in Arabidopsis thaliana. Development.

[16] Takahashi Y., Soyano T., Kosetsu K., Sasabe M., Machida Y. (2010). HINKEL kinesin, ANP MAPKKKs and MKK6/ANQ MAPKK, which phosphorylates and activates MPK4 MAPK, constitute a pathway that is required for cytokinesis in Arabidopsis thaliana. Plant Cell Physiol..

[17] Yang C.Y., Spielman M., Coles J.P., Li Y., Ghelani S., Bourdon V., Brown R.C., Lemmon B.E., Scott R.J., Dickinson H.G. (2003). TETRASPORE encodes a kinesin required for male meiotic cytokinesis in Arabidopsis. Plant J..

[18] Zeng Q.N., Chen J.G., Ellis B.E. (2011). AtMPK4 is required for male-specific meiotic cytokinesis in Arabidopsis. Plant J..

[19] Cromer L., Heyman J., Touati S., Harashima H., Araou E., Girard C., Horlow C., Wassmann K., Schnittger A., De Veylder L. (2012). OSD1 promotes meiotic progression via APC/C inhibition and forms a regulatory network with TDM and CYCA1;2/TAM. PLoS Genet..

[20] Azumi Y., Liu D., Zhao D., Li W., Wang G., Hu Y., Ma H. (2002). Homolog interaction during meiotic prophase I in Arabidopsis requires the SOLO DANCERS gene encoding a novel cyclin-like protein. EMBO J..

[21] De Storme N., Copenhaver G.P., Geelen D. (2012). Production of diploid male gametes in Arabidopsis by cold-induced destabilization of postmeiotic radial microtubule arrays. Plant Physiol..

[22] Andreuzza S., Nishal B., Singh A., Siddiqi I. (2015). The chromatin protein DUET/MMD1 controls expression of the meiotic gene TDM1 during male meiosis in Arabidopsis. PLoS Genet..

[23] Wang J., Niu B., Huang J., Wang H., Yang X., Dong A., Makaroff C., Ma H., Wang Y. (2016). The PHD Finger Protein MMD1/DUET Ensures the Progression of Male Meiotic Chromosome Condensation and Directly Regulates the Expression of the Condensin Gene CAP-D3. Plant Cell.

[24] Reddy T.V., Kaur J., Agashe B., Sundaresan V., Siddiqi I. (2003). The DUET gene is necessary for chromosome organization and progression during male meiosis in Arabidopsis and encodes a PHD finger protein. Development.

[25] Yang X., Makaroff C.A., Ma H. (2003). The Arabidopsis MALE MEIOCYTE DEATH1 gene encodes a PHD-finger protein that is required for male meiosis. Plant Cell.

[26] Liu Z., Makaroff C.A. (2006). Arabidopsis separase AESP is essential for embryo development and the release of cohesin during meiosis. Plant Cell.

[27] Li Y., Shen Y., Cai C., Zhong C., Zhu L., Yuan M., Ren H. (2010). The type II Arabidopsis formin14 interacts with microtubules and microfilaments to regulate cell division. Plant Cell.

[28] Dong X., Hong Z., Sivaramakrishnan M., Mahfouz M., Verma D.P.S. (2005). Callose synthase (CalS5) is required for exine formation during microgametogenesis and for pollen viability in Arabidopsis. Plant J..

[29] Lu P., Chai M., Yang J., Ning G., Wang G., Ma H. (2014). The Arabidopsis CALLOSE DEFECTIVE MICROSPORE1 gene is required for male fertility through regulating callose metabolism during microsporogenesis. Plant Physiol..

[30] Ravi M., Marimuthu M.P.A., Siddiqi I. (2008). Gamete formation without meiosis in Arabidopsis. Nature.

[31] Enns L.C., Kanaoka M.M., Torii K.U., Comai L., Okada K., Cleland R.E. (2005). Two callose synthases, GSL1 and GSL5, play an essential and redundant role in plant and pollen development and in fertility. Plant Mol. Biol..

[32] Brownfield L., Yi J., Jiang H., Minina E., Twell D., Köhler C. (2015). Organelles maintain spindle position in plant meiosis. Nat. Commun..

[33] Liu M., Nadar V.C., Kozielski F., Kozlowska M., Yu W., Baas P.W. (2010). Kinesin-12, a Mitotic Microtubule-Associated Motor Protein, Impacts Axonal Growth, Navigation, and Branching. J. Neurosci..

[34] Oh S., Bourdon V., Dickinson H., Twell D., Park S. (2014). Arabidopsis Fused kinase TWO-IN-ONE dominantly inhibits male meiotic cytokinesis. Plant Reprod..

[35] Kosetsu K., Matsunaga S., Nakagami H., Colcombet J., Sasabe M., Soyano T., Takahashi Y., Hirt H., Machida Y. (2010). The MAP kinase MPK4 is required for cytokinesis in Arabidopsis thaliana. Plant Cell.

[36] Chaudhury A., Craig S., Dennis E., Lavithis M., Taylor P., Singh M., Knox R., Signer E. (1994). Genetic control of male fertility in Arabidopsis thaliana: Structural analysis of premeiotic developmental mutants. Sex. Plant Reprod..

[37] Liu B., De Storme N., Geelen D. (2017). Gibberellin induces diploid pollen formation by interfering with meiotic cytokinesis. Plant Physiol..

[38] Millar A.A., Gubler F. (2005). The Arabidopsis GAMYB-like genes, MYB33 and MYB65, are microRNA-regulated genes that redundantly facilitate anther development. Plant Cell.

[39] d’Erfurth I., Jolivet S., Froger N., Catrice O., Novatchkova M., Mercier R. (2009). Turning Meiosis into Mitosis. PLoS Biol..

[40] Zamariola L., De Storme N., Vannerum K., Vandepoele K., Armstrong S.J., Franklin F.C.H., Geelen D. (2014). SHUGOSHINs and PATRONUS protect meiotic centromere cohesion in Arabidopsis thaliana. Plant J..

[41] Yang X., Boateng K.A., Yuan L., Wu S., Baskin T.I., Makaroff C.A. (2011). The Radially Swollen 4 separase mutation of Arabidopsis thaliana blocks chromosome disjunction and disrupts the radial microtubule system in meiocytes. PLoS ONE.

[42] Zamariola L., De Storme N., Tiang C., Armstrong S.J., Franklin F.C.H., Geelen D. (2013). SGO1 but not SGO2 is required for maintenance of centromere cohesion in Arabidopsis thaliana meiosis. Plant Reprod..

[43] De Storme N., Keceli B.N., Zamariola L., Angenon G., Geelen D. (2016). CENH3-GFP: A visual marker for gametophytic and somatic ploidy determination in Arabidopsis thaliana. BMC Plant Biol..

[44] Liu B., De Storme N., Geelen D. (2018). Cold-induced male meiotic restitution in Arabidopsis thaliana is not mediated by GA-DELLA signaling. Front. Plant Sci..

[45] De Storme N., Zamariola L., Mau M., Sharbel T.F., Geelen D. (2013). Volume-based pollen size analysis: An advanced method to assess somatic and gametophytic ploidy in flowering plants. Plant Reprod..

[46] Brownfield L., Hafidh S., Borg M., Sidorova A., Mori T., Twell D. (2009). A plant germline-specific integrator of sperm specification and cell cycle progression. PLoS Genet..

[47] Wang J., Yu C., Zhang S., Ye J., Dai H., Wang H., Huang J., Cao X., Ma J., Ma H. (2020). Cell-type-dependent histone demethylase specificity promotes meiotic chromosome condensation in Arabidopsis. Nat. Plants.

