# Supplementary material for: A Hypomorphic Mutant of PHD Domain Protein Male Meiocytes Death 1
Source: Genes (Basel). 2021 Apr 1;12(4):516. doi: 10.3390/genes12040516 (PMC8066392; doi:10.3390/genes12040516)
Supplement: Supplementary file 1 [file genes-12-00516-s001.pdf]

A Hypomorphic Mutant of PHD Domain Protein Male Meiocytes Death 1

Bing Liu[1, 2*], Chunlian Jin[2], Nico De Storme[2, 3], Sébastien Schotte[2], Cédric Schindfesse[2], Tim De Meyer[4], and Danny Geelen[2*]

[1]Current address of the first author: College of Life Sciences, South-Central University for Nationalities, 430074 Wuhan, China;

[2]Address where most of the experiments were performed: Department of Plants and Crops, unit HortiCell, Faculty of Bioscience Engineering, Ghent University, Coupure links, 9000 Ghent, Belgium;

[3]Division of Crop Biotechnics, Department of Biosystems, KU Leuven, 3001 Leuven, Belgium;

[4]Department of Data analysis and mathematical modelling, Ghent University, Coupure links, 9000 Ghent, Belgium;

*To whom correspondence should be addressed:

Bing Liu (bl472@scuec.edu.cn);

Danny Geelen (senior author, Danny.Geelen@UGent.be).

Supplemental figures

Supplemental figure S1.

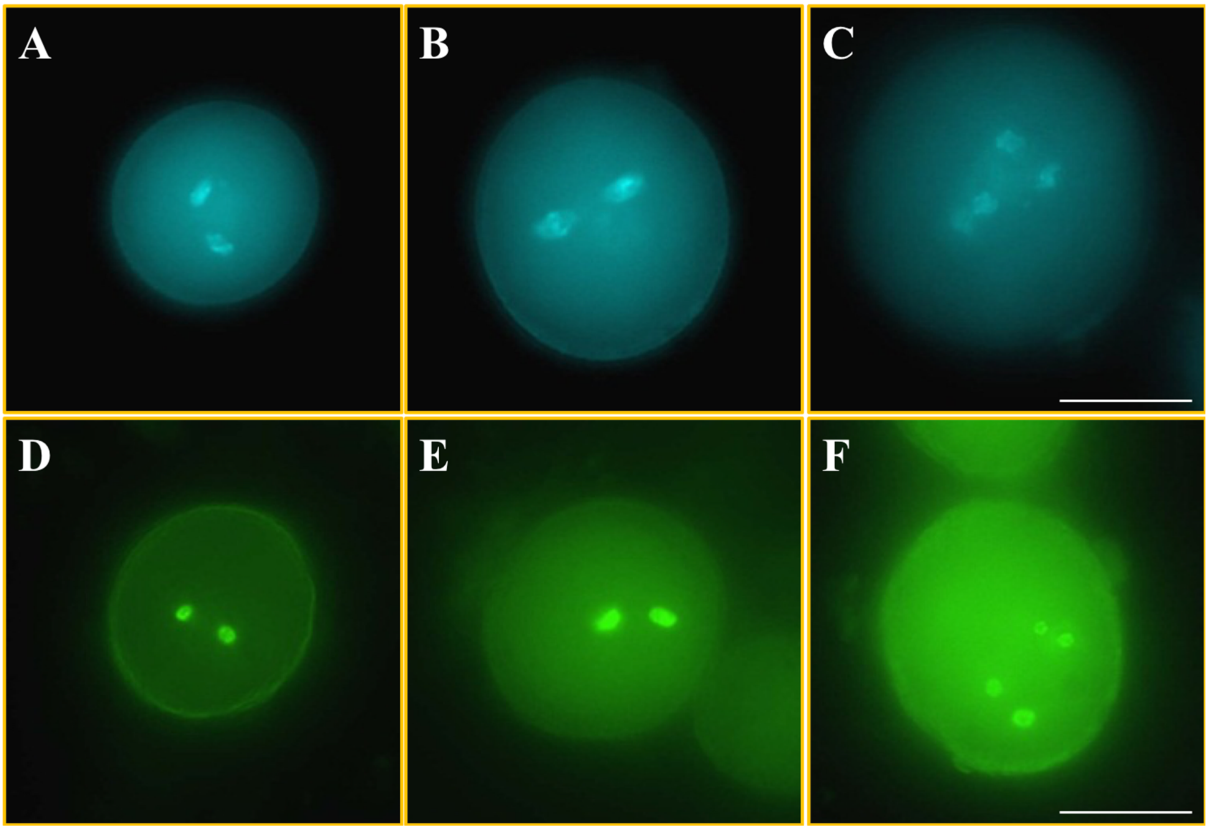

Supplemental figure S1. *mmr1* produces enlarged pollen grains with increased DNA content. A and D, DAPI-stained (A) and *pMGH3::H2B-GFP* expressing (D) haploid pollen grains from wild-type Col-0 plants. B and C, DAPI-stained enlarged pollen grains from *mmr1* plants. E and F, *pMGH3::H2B-GFP* expressing enlarged pollen grains from *mmr1* plants. Scale bar = 10 µm.

Supplemental figure S2.

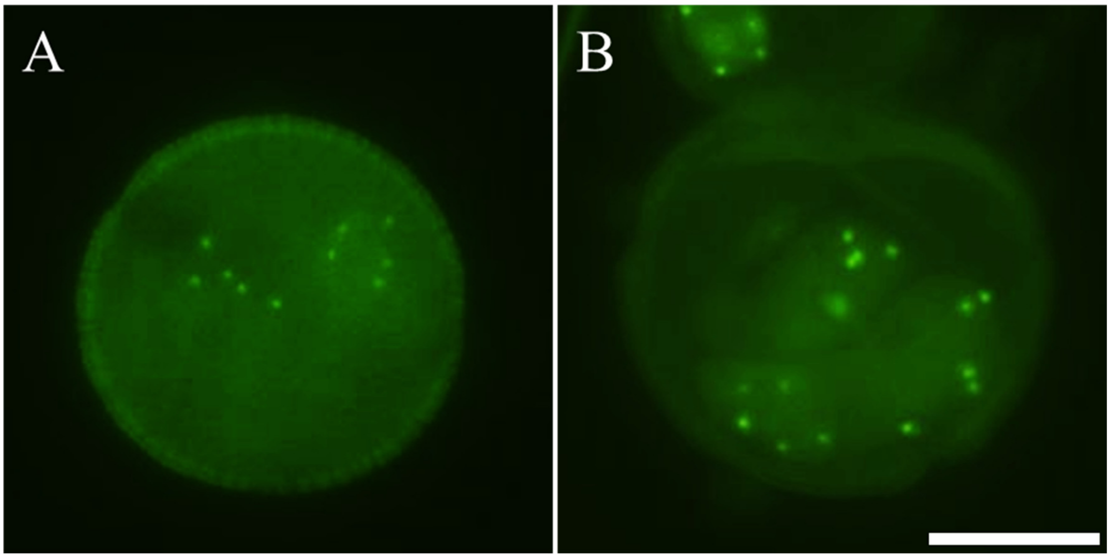

Supplemental figure S2. *mmr1* produces diploid or polyploid microspores. A and B, Diploid (A) and triploid (B) microspores expressing *pWOX2::CENH3-GFP* in *mmr1*. Scale bar = 10 µm.

Supplemental figure S3.

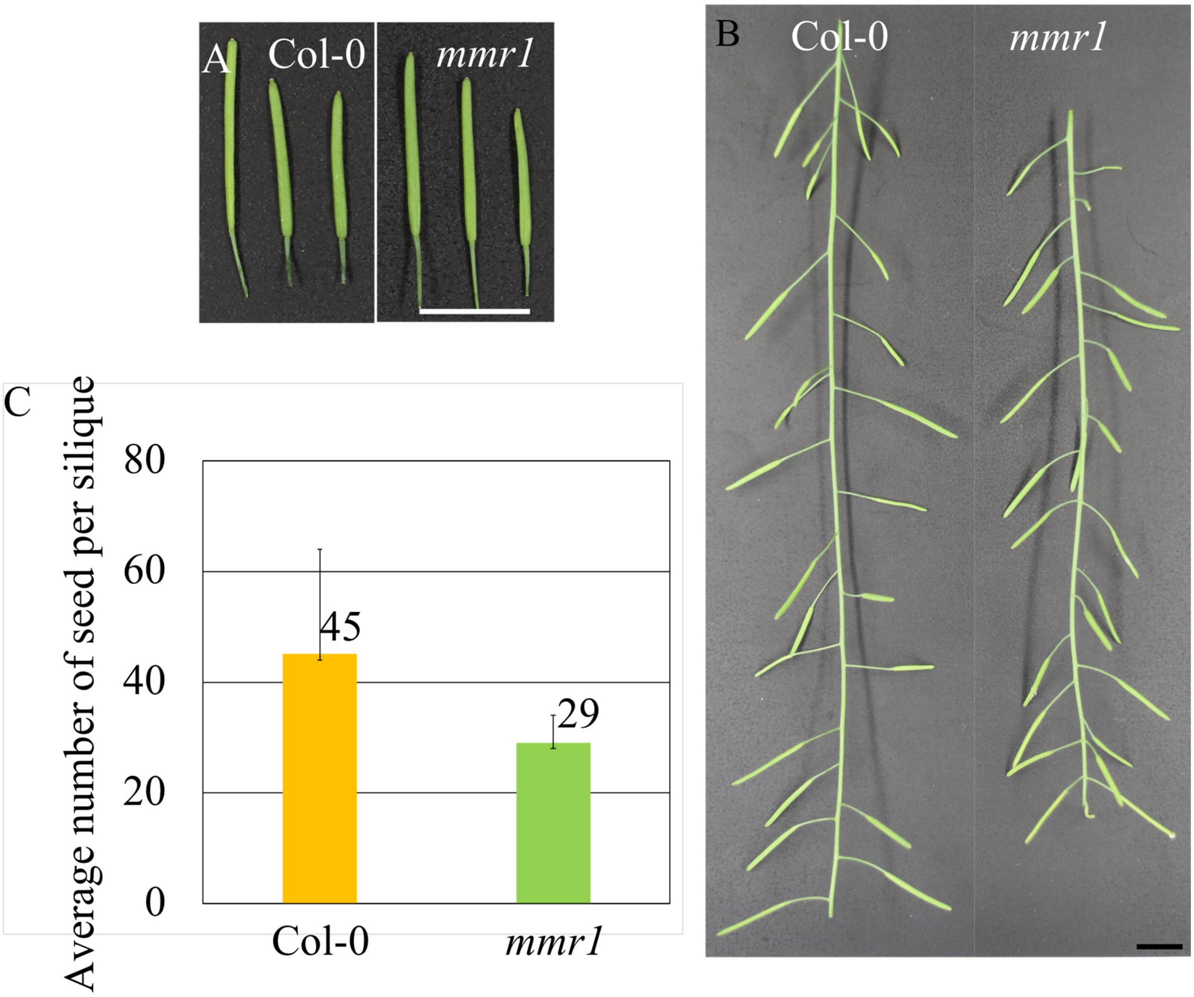

Supplemental figure S3. *mmr1* displays a mildly reduced fertility. A and B, *mmr1* plants display normal silique development. Scale bar = 1 cm. C, Histogram showing the average number of seeds per silique. Error bars represent standard deviation of the mean values. Three biological repeats were analyzed, and for each plant individual, three siliques were analyzed.

Supplemental figure S4.

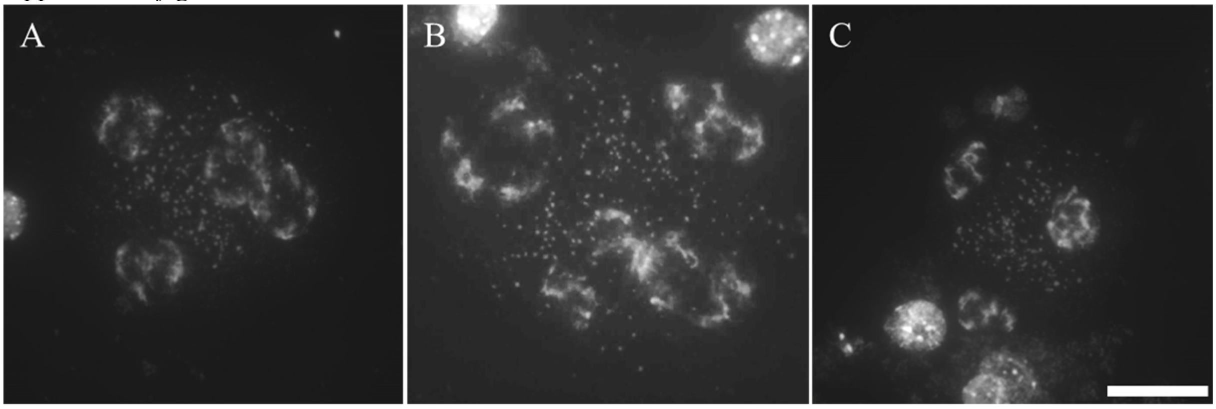

Supplemental figure S4. Tetrad stage meiocytes in the *mmr1* mutant stained by DAPI. A-C, Co-localization of nuclei in the *mmr1* tetrad. Scale bar = 10 µm.

Supplemental figure S5.

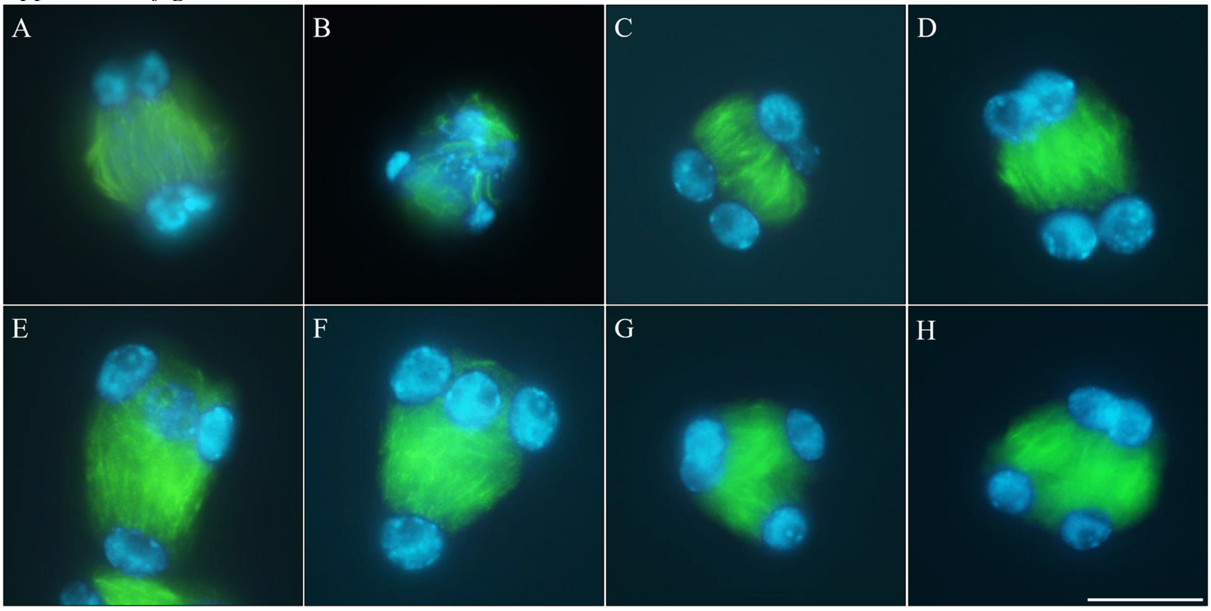

Supplemental figure S5. *mmr1* exhibits defective phragmoplast and RMA. A-F, RMA at tetrad stage displaying balanced-dyad (A and B), unbalanced-dyad (C and D) and triad (E and F) in the *mmr1* mutant. G and H, Telophase II stage meiocytes displaying parallel (G) and tripolar (H) phragmoplast in the *mmr1* mutant. Scale bar = 10 µm.

Supplemental figure S6.

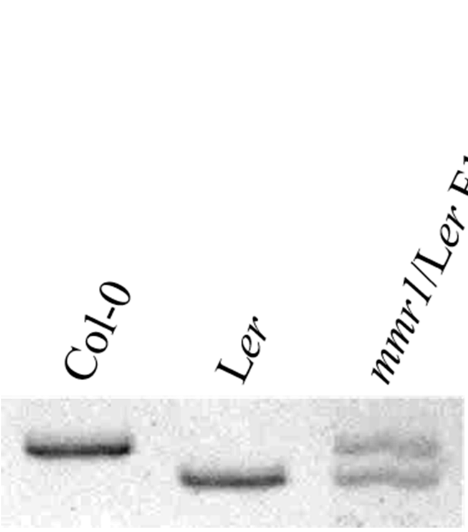

Supplemental figure S6. Genotyping of F1 individual obtained by crossing between homozygous *mmr1* mutant and wild-type L*er* plants using SSLP marker primer F3L12.

Supplemental figure S7.

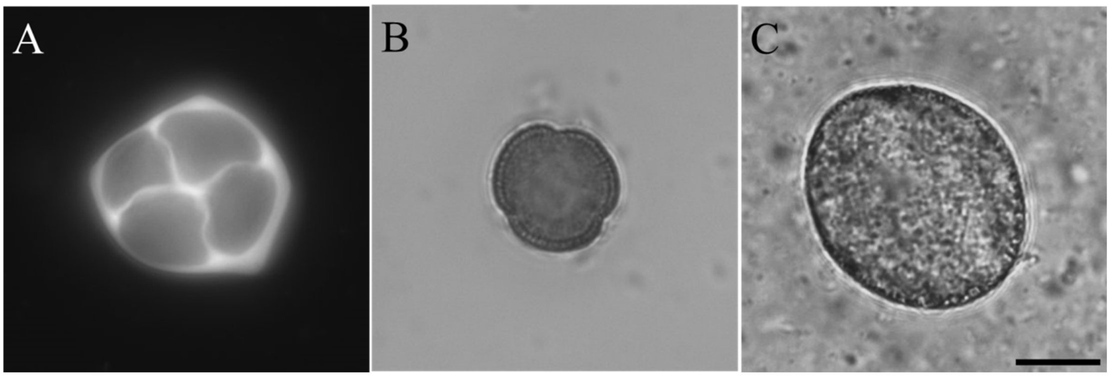

Supplemental figure S7. Phenotyping of F1 plants obtained by crossing homozygous *mmr1* mutant with wild-type L*er* plants. A-C, Normal tetrad with regular meiotic cytokinesis (A), haploid unicellular stage microspore (B) and haploid mature pollen (C). Scale bar = 10 µm.

Supplemental figure S8.

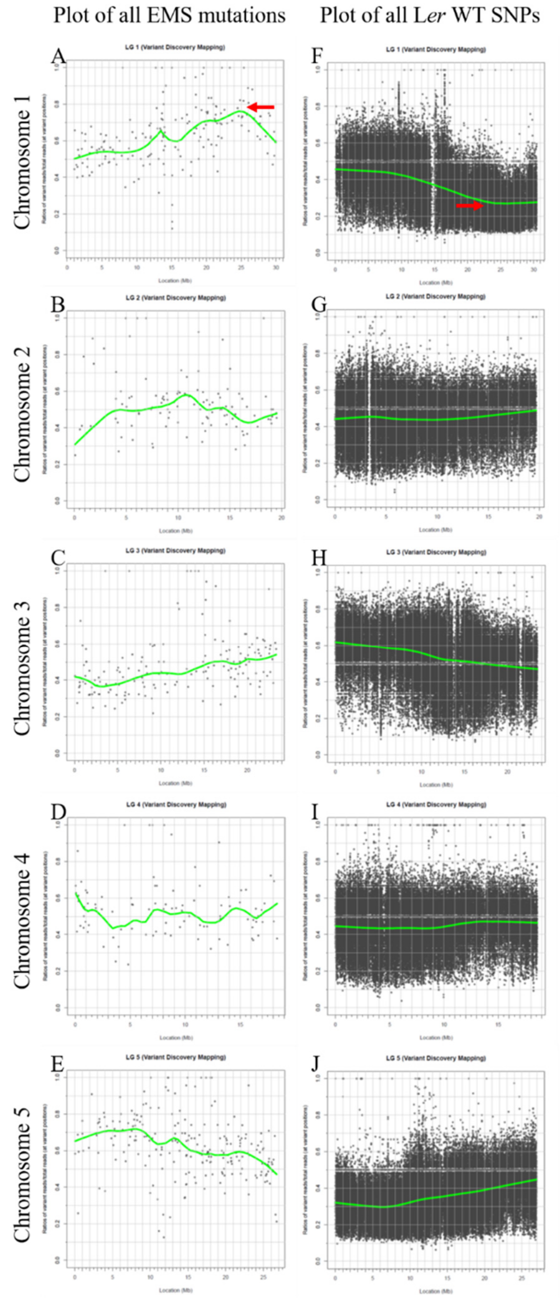

Supplemental figure S8. Whole genome sequencing analysis of pooled *mmr1*/L*er* F2 plants showing the large pollen phenotype. An F2 population was generated by crossing *mmr1* and L*er*. A-E, Frequency plot of variant sequence reads for the five chromosomes. The green lines represent ratio of variant sequence reads over total reads. At the bottom of chromosome 1, at region close to 25Mb, around 75% of total reads contain an EMS mutation. F-J, Frequency plots of sequence reads corresponding to L*er* sequences carrying a single nucleotide polymorphisms (SNPs) on the 5 chromosomes.

Supplementary Tables

Supplemental Table S1. Primer for genotyping of F1 progenies (*mmr1* crossing with wild-type L*er*).

Primer	genotyping primer sequence (5' to 3')	AT (°C)
F3L12 F	TCC AAT CAA ACA TAA ATT AGT CAC TC	46
F3L12 R	GTA AGT TTA AGG TTT TCA CAC ACG	

Supplementary Table S2. Quantification of *mmr1* meiotic restitution by monitoring microtubules.

	Prophase I	M I	A I	Interkinesis	M II	T II		Tetrad			
						Normal	Abnormal	Balanced-dyad	Unbalanced-dyad	Triad	Tetrad
Col-0	23	44	25	20	17	4	0	0	0	4 (6.45%)	58 (93.55%)
mmr1	30	13	29	31	14	3 (25%)	9 (75%)	16 (7.84%)	8 (3.92%)	74 (36.27%)	106 (51.96%)

Supplemental Table S3. Mutation of *mmr1* at the *MMD1*/*DUET* locus. *mmr1* contains a C to T transition mutation in the third exon of *MMD1*/*DUET* (at site 2168 bp), leading to an alteration of codon GGC to GAC at site 1854 bp of *MMD1*/*DUET* mRNA, which consequently causes a replacement of Gly (G) by Asp (D), at the 618th amino acid position of the MMD1/DUET protein.

	Sequence		Site in gene/protein sequence
	MMD1/DUET	*mmr1*	
gDNA	C**C**G	C**T**G	2168
mRNA	G**G**C	G**A**C	1854
Amino acids	Gly (**G**)	Asp (**D**)	618
